# Aortic Valve Repair and Early-Career Surgeons—Nothing Is Impossible

**DOI:** 10.3390/jcdd10070284

**Published:** 2023-07-01

**Authors:** Anze Djordjevic, Igor Rudez

**Affiliations:** 1 Department of Cardiac Surgery, University Medical Centre Maribor, 2000 Maribor, Slovenia; 2 Department of Cardiac and Transplant Surgery, University Hospital Dubrava, 10000 Zagreb, Croatia

Aortic valve repair with either the reimplantation of the aortic valve or aortic root remodelling with the external annuloplasty procedure is the most effective means of treating aortic regurgitation and/or aortic root aneurysms. Because a large body of evidence supports both the effectiveness and safety of AV reconstruction, the most recent 2021 European Society of Cardiology/European Association of Cardiothoracic Surgery Guidelines for the management of valvular heart disease endorse its application in suitable patients as a Class IIb recommendation for severe aortic insufficiency and as a Class I recommendation for aortic root aneurysms [1]. Yet, the procedure is presently underused.

How can we explain this widespread failure to treat isolated AV regurgitation and/or aortic root aneurysms? The procedure is undoubtedly complex and requires a thorough theoretical as well as practical knowledge from the whole heart team, especially the primary surgeon. Early-career surgeons, who wish to start their own AV repair program, should be familiar with the anatomy and (patho)physiology of the aortic root with the knowledge gained from both cadaveric specimens [2], echocardiography [3], and novel imaging modalities, such as computer tomography reconstructions [4,5]. Perhaps even more important than with mitral valve repair is a sufficient understanding of the mechanisms leading to aortic root pathology; this is imperative so that one can identify suitable candidates among their patients to achieve durable AV repair.

In regard to surgical experience, an early-career surgeon embarking on this journey should be able to perform a straightforward aortic valve replacement with no difficulties and have performed at least twenty composite valve-graft conduit root replacements. Comprehensive and thorough knowledge of all the surgical steps of either full aortic root replacement or isolated AV repair in both tricuspid and bicuspid valves could and should be obtained from in-depth articles, such as the *How I Teach It* series from *Annals of Thoracic Surgery* [6,7,8,9] and further deepened through hands-on courses and interactive surgical atlases. 

Nevertheless, the most effective way of preparing oneself for AV repair is more close contact with an experienced colleague in a repair-oriented centre of excellence (for example Paris, Toronto or New York) who can explain concepts and help to minimize the learning curve that is to be expected. This is true especially when it comes down to specific situations or failures. Is the present fenestration too large or too near the commissure to obtain long-term repair durability? Are present (non-obstructing) calcifications (of only one leaflet) precluding safe repair? Are the coronary ostia too high, i.e., too close to the sinotubular junction to prevent reconstructing only the AV in case of isolated regurgitation and thus requiring full aortic root replacement? When exactly is the effective height sufficient when measuring it with a dedicated calliper?

It is the authors’ opinion that at the beginning of one’s AV repair journey, root remodelling with an external annuloplasty procedure is most suitable for a number of reasons. The first is related to superior haemodynamics with regard to the formation of vortical flow with preserved root expansibility and more physiological valve movements. Second, it is a highly standardised and reproducible technique with no “artistic” decisions (requiring decades of experience) along the way. Third, the fallback option in full root replacement if there is persistent insufficiency is to re-clamp and perform a straightforward AV replacement, which is difficult in the AV reimplantation technique. 

Another very important aspect of failure to perform more AV repairs is the lack of long-term results. However, this is changing as we speak. With their investigation of aortic valve repair in patients with connective tissue disease operated electively for root aneurysm < 60 mm with aortic regurgitation (AR) < 1/4, Van Hoof and colleagues [10] inform us that we should perform AV repair in these patients. In their analysis of 80 patients published in *Heart*, 5-year survival reached 99% with no Stanford type A dissections and 92% freedom from significant postoperative aortic regurgitation. This important article has set the standards for the optimal care of these patients. It is an upgrade on previously published results both in the dystrophic aortic insufficiency population [11] as well as in heritable aortic disorders [12]. 

Aortic valve repair is dramatically underperformed. In 2023, there are fewer and fewer reasons not to perform an AV repair in suitable patients with either aortic root aneurysm or isolated aortic insufficiency and thus prevent them from structural valve degeneration, significant bleeding, or thromboembolic events, as seen in AV replacement. Learning from one’s own failures and ensuring proper follow-up should additionally strengthen every AV repair program, this fact being extremely crucial for an early-career surgeon. With the introduction of multicentre surgical registries [13], this goal is achievable now more than any other time in the past.
